# Nativity is associated with sugar-sweetened beverage and fast-food meal consumption among mexican-origin women in Texas border *colonias*

**DOI:** 10.1186/1475-2891-10-101

**Published:** 2011-09-30

**Authors:** Joseph R Sharkey, Cassandra M Johnson, Wesley R Dean

**Affiliations:** 1 Program for Research in Nutrition and Health Disparities, School of Rural Public Health, Texas A&M Health Science Center, College Station, TX (USA; 2 UNC Center for Health Promotion and Disease Prevention and Department of Nutrition, UNC Gillings School of Global Public Health, Chapel Hill, NC (USA

## Abstract

**Background:**

Trends of increasing obesity are especially pronounced among Mexican-origin women. There is little understanding of dietary patterns among U.S.- and Mexico-born Mexican-origin individuals residing in new-destination immigrant communities in the United States, especially behaviors related to obesity, such as consumption of sugar-sweetened beverages (SSB) and fast-food meals (FFM).

**Methods:**

The study used survey data of 599 adult Mexican-origin women from the 610 women who completed the 2009 *Colonia *Household and Community Food Resource Assessment (C-HCFRA), which was completed in person by trained *promotora*-researchers in 44 *colonias *near the Texas border towns of Progreso and La Feria. Data included demographic characteristics (age, education, nativity or country of birth, household income, household composition, and employment status), access to transportation, self-reported height and weight, food and nutrition assistance program participation, and consumption of SSB and FFM. Descriptive statistics were calculated by nativity (U.S.-born vs. Mexico-born); multivariable linear regression models were estimated for correlates of consumption of SSB and FFM.

**Results:**

There are three major findings related to nativity. First, U.S.-born women consumed more SSB and FFM than Mexican-born counterparts in the same areas of *colonias*. Second, in the combined sample and controlling for other population characteristics, being born in Mexico was independently associated with FFM (fewer FFM), but not with SSB. Third, in analyses stratified by nativity, FFM and SSB were associated with each other among both nativity groups. Among Mexico-born women only, age, presence of a child, or being a lone parent was significantly associated with SSB; full-time employment, being a lone parent, and SSB consumption were each independently associated with increased frequency of FFM.

**Conclusions:**

Our analyses revealed differences in prevalence and correlates of SSB and FFM based on country of birth. Nativity, as a proxy for acculturation, may indicate the extent that immigrants have adopted behaviors from their new environment. However, nativity could also indicate limited accessibility to resources such as food/nutrition assistance programs, transportation, and proper documentation. Additionally, future research should focus on expanding our understanding of the meaning of nativity among individuals who share common contextual factors, but may have different life course experiences and resources needed to transition into a new place. Additional measures should be considered such as educational and occupational background, migration history, documentation status, and dietary acculturation, which may better explain heterogeneity within Hispanic subgroups.

## Background

Trends of increasing obesity are especially pronounced among Mexican-origin adults, who tend to be more overweight and obese than non-Hispanic white adults, especially Mexican-origin women who are one of the most obese populations in the U.S. [1,2]. This is of great concern for several reasons. First, since the 2000 U.S. Census, there has been a dramatic increase in the Hispanic or Latino population [3], primarily of Mexican origin [4]. Although much of this growth has occurred in traditional gateway locations such as Chicago, or the U.S. border with Mexico, nearly half of the U.S. population of Hispanics lives outside traditional gateway states [5]. Concentrations of Mexican immigration can be found in new destination immigrant settlements in the Midwest, the South, the Northeast, and the Northwest [5-11]. Second, obesity is related to other comorbidities including diabetes, which also is more common in Mexican-origin women than African American or non-Hispanic white women [12]. Third, there is little understanding of dietary patterns among Mexican-origin individuals in the United States, especially behaviors related to obesity [13-16], such as consumption of sugar-sweetened beverages (SSB) and fast-food meals (FFM) [17,18].

In order to provide national guidance for an overall healthy diet, the 2010 Dietary Guidelines for Americans (DGA) includes a recommendation that individuals reduce intake of calories from added sugars and fats to maintain energy balance, meet nutrient requirements, and reduce risk for common chronic diseases including obesity and diabetes [19]. This recommendation provides motivation for understanding dietary behaviors associated with intake of these "empty calories", namely consumption of SSB and FFM [17,20-23]. Prior work also demonstrates the positive correlation between consumption of unhealthy beverages and unhealthy foods [24], and more specifically the consumption of SSB and FFM [25].

Research findings suggest that Mexican-origin adults, both U.S.- and foreign-born, may be more at risk for diet-related chronic diseases [1,2,12,26-30] and obesogenic dietary behaviors including intake of SSB and FFM [13-16]. Among Mexican-origin adults, greater acculturation to foods and food patterns in the United States was associated with less-healthy dietary behaviors [31-34]. Acculturation is a multidimensional process affected by country of birth, age of arrival, years living in the United States, and contextual factors such as neighborhood composition, regional history of migration, and social networks [33,35-37]. However, there is a paucity of research devoted to understanding dietary behaviors relevant to chronic disease, such as SSB and FFM consumption among Mexican-origin immigrant women in the U.S, especially those living in areas that attract Mexican immigrants such as settlements or *colonias *in the border region between the U.S. and Mexico or the many new destination Mexican settlements spread throughout the U.S. [33,38-40]. In addition, the literature offers mixed findings regarding the influence of country of birth on health behaviors or health status [15,33,41-47]. Rather, most studies of this population of Mexican-origin women have focused on women's health (reproductive health, pregnancy, maternal and child health, birth outcomes and breast cancer) [6,42,48-54], intimate partner relationships including abuse [55,56], substance abuse [57], stress/mental health [58-61], or focused on their children [62,63]. One recent study did find a greater degree of food insecurity among Mexican-born women, compared with U.S.-born women in the same *colonias *[64]. Despite an increased risk for diet-related chronic diseases, there are only a handful of studies that describe dietary patterns of Mexican-origin women [16,27,39]. Of these, one examined dietary habits to include FFM [40], and none focused on understanding both SSB and FFM consumption, particularly the extent of daily consumption of SSB and weekly frequency of FFM, or the factors associated with SSB and FFM among the hard-to-reach Mexican-origin families who reside in the growing *colonias *along the Texas border with Mexico. Research suggests that geographic location (greater access to convenience stores and fast food restaurants) [65-70], household composition (children in the home) [25], and individual or family characteristics (age, employment status, household resources) [25,71-74] were associated with unhealthy eating behaviors, such as SSB and FFM [75-78]. Considering that sociocultural, physical and economic elements of the environment may increase SSB and FFM, the purpose of this study was to examine data from face-to-face interviews conducted in Spanish by specially-trained *promotora*-researchers (indigenous community health workers trained in research methods) in 44 *colonias *along the Texas-Mexico border to: 1) compare demographic characteristics and eating behaviors between women born in the U.S. and women born in Mexico, all living in the same *colonias *and with similar spatial access to food resources, 2) examine the relation of demographic characteristics and eating behaviors to SSB and FFM, and 3) determine if the relationships differed based on nativity.

## Methods

### Participants

The study used survey data of 599 adult, Mexican-origin women from the 610 women who completed the 2009 *Colonia *Household and Community Food Resource Assessment (C-HCFRA), which was conducted in 44 *colonias *near the Texas border towns of Progreso and La Feria and has been previously described [64]. Eleven women were excluded from analysis for missing data on daily consumption of SSB and/or weekly frequency of FFM.

### Data Collection

Four *promotora*-researchers, who received special training, recruited participants door-to-door on weekdays and weekend days from September to October 2009, acquired informed consent, conducted face-to-face surveys in Spanish, and disbursed a modest incentive ($5) at the completion of the survey. Details of the training of the *promotora*-researchers, recruitment of participants, and development and modification of the survey instrument to ensure semantic and conceptual equivalence and social and cultural appropriateness have been described elsewhere [64]. All data were collected in Spanish; all *promotora*-researchers were native Spanish speakers who also resided in nearby *colonias*.

### Measures

*Demographic characteristics *included age, education, ethnic self-identification, marital status, nativity (country of birth), household composition (number of adults and children in the household), household income, and employment status. Federal poverty level (FPL) for 2009 was calculated from household income and composition data using 2009 Federal Poverty Guidelines [79]. Self-reported height and weight were used to calculate body mass index (BMI) in kg/m^2^. Categories of BMI were constructed as normal (< 25 kg/m^2^), overweight (25-29.9 kg/m^2^), and obese (≥ 30 kg/m^2^). *Access to transportation *was assessed through car ownership, car availability, and source of transportation. *Food and nutrition assistance programs *included four federal programs: 1) Supplemental Nutrition Assistance Program (SNAP), 2) Women, Infants, and Children (WIC), 3) School Breakfast Program (SBP), and 4) National School Lunch Program (NSLP).

*Eating behaviors *were measured by self-reported daily servings of fruit and vegetables, SSB, and weekly frequency of FFM. To capture SSB and FFM consumption, participants were asked: "How many cans or glasses of regular soda (not diet) or sugar-sweetened beverages do you drink on an average day?" and "How many times a week do you eat fast food meals?" These measures were previously used in community-based work in North Carolina [80-82]. Two questions from a validated, self-reported two-item screener were combined to describe fruit and vegetable intake [83,84].

### Statistical Analysis

Release 11 of Stata Statistical Software (College Station, TX) was used for all statistical analyses; *p *< 0.05 was considered statistically significant. Descriptive statistics were estimated for demographic characteristics, transportation, food and nutrition program participation, and eating behaviors. Comparisons were made between women who were born in the United States and in Mexico using *χ^2 ^*test (categorical variables) and *t*-test (continuous variables). A conservative Bonferroni correction (alpha rejection region/number of tests to be conducted) was used to reduce Type I error rate for each individual test from 0.05 to 0.002 [85]. Bivariate correlations between SSB, FFM, and demographic characteristics, food and nutrition program participation, and eating behaviors were estimated. Separate linear regression models were estimated for the entire sample and stratified by nativity (U.S.-born and Mexico-born) to determine the association of independent variables with SSB and FFM. Statistically significant (*p *< 0.05) variables for demographic characteristics, transportation, food and nutrition program participation, and eating behaviors from the bivariate correlation estimations were simultaneously entered; backward elimination strategy was used, which sequentially removed statistically non-significant variables, to obtain the "best" set of independent variables [85]. Adjusted coefficients and standard errors (SE) are reported.

## Results

Table 1 presents descriptive statistics for the entire sample and differences in proportions between women who were born in the United States (32.4%, *n *= 194) and those born in Mexico (67.6%, *n *= 405). Compared with U.S.-born women, a greater proportion of Mexico-born women completed less than a 7^th ^grade education, reported a household income at or below 75% of the FPL, were married, had a greater number of children living in the household, were overweight, and did not have a car available. A greater proportion of U.S.-born women were employed full-time outside the home or were lone parents. Although similar proportions of U.S.- and Mexico-born women reported no consumption of SSB (21.1% of U.S.-born and 21.2% of Mexico-born), U.S.-born women consumed a greater number of cans/glasses of SSB each day (1.9 vs. 1.6); and among women who consumed at least one can/glass of SSB, U.S.-born women reported 2.4 ± 1.9 compared with 2.0 ± 1.3 for Mexico-born (*p *= 0.004). U.S.-born women also consumed more FFM each week (1.4 vs. 0.9); and among women who consumed ≥ 1 FFM (72.7% of U.S.-born vs. 64.9% of Mexico-born, *p *= 0.058), U.S.-born women reported 2.0 ± 1.4 FFMs/week compared with 1.4 ± 0.7 for Mexico-born women (*p *< 0.001). Eight of the comparisons remained significant after adjusting for multiple comparisons with a revised level of statistical significance (*p *≤ 0.002). In bivariate correlations (data not shown), the following characteristics were significantly correlated with FFM and not with SSB: obesity (*r *= - 0.13), full-time employment (*r *= 0.17), household income > 100% FPL (*r *= 0.18), number of children in the household (*r *= - 0.09), SNAP participant (*r *= - 0.13), and not having a car available (*r *= - 0.08); other variables were correlated with both FFM and SSB. In addition SSB was correlated with FFM (*r *= 0.29, *p *< 0.001).

**Table 1:** Difference in Demographic Characteristics, Food and Nutrition Program Participation, and Eating Behaviors between U.S.-Born and Mexico-Born Mexican-origin Women in Texas-Mexico Border *Colonias *(*n *= 599)^1^

	*Total Sample (n = 599)*	*U.S.-born (n = 194)*	*Mexico-born (n = 405)*
*Variable*	*% (n)*	*% (n)*	*% (n)*
***Demographic characteristics***			
Age, y (mean ± SD)	39.9 ± 14.5	38.1 ± 14.7	40.8 ± 12.3*
Education			
< 7^th ^grade	30.2 (181)	10.8 (21)	39.5 (160)***^2^
7^th^-11^th ^grade	31.5 (189)	24.7 (48)	34.8 (141)**
Ethnic self-identification			
Hispanic	9.2 (55)	19.6 (38)	4.2 (17)***^2^
Mexican American	27.9 (167)	76.3 (148)	4.7 (19)***^2^
Mexican	61.8 (370)	1.5 (3)	90.6 (367)***^2^
Household income (FPL)			
No response	25.4 (152)	26.8 (52)	24.7 (100)
≤ 75% FPL	63.8 (382)	55.1 (107)	67.9 (275)**^2^
76%-100% FPL	8.5 (51)	12.4 (24)	6.7 (27)**
Employment			
Full-time outside home for wages	26.7 (160)	33.5 (65)	23.5 (95)**
Part-time outside home	21.4 (128)	19.1 (37)	22.5 (91)
Marital status			
Married	59.8 (358)	51.0 (99)	63.9 (259)**
Household composition			
Adults (total)	1.9 ± 0.7	1.9 ± 0.8	2.0 ± 0.7
Lone parent	19.9 (119)	27.8 (54)	16.0 (65)***^2^
Children (total)	2.0 ± 1.6	1.7 ± 1.4	2.1 ± 1.7**
Children (≥ 1)	79.5 (476)	77.3 (150)	80.5 (326)
Total adults and children	3.9 ± 1.8	3.6 ± 1.7	4.1 ± 1.9**
BMI (kg/m^2^)^3^			
Normal (< 25)	30.3 (177)	35.1 (67)	28.0 (110)
Overweight (25-29.9)	35.1 (205)	26.2 (50)	39.4 (155)***^2^
Obese (≥ 30)	34.6 (202)	38.7 (74)	32.6 (128)
Transportation			
No car available	19.5 (117)	11.9 (23)	23.2 (94)***^2^
Ride with family or friend^4^	88.9 (104)	91.3 (21)	88.3 (83)
Pay for transportation^4^	29.1 (34)	26.1 (6)	29.8 (28)
***Food and nutrition program***			
SNAP	55.1 (330)	55.7 (108)	54.8 (222)
WIC^5^	42.6 (203)	42.7 (64)	42.6 (139)
School breakfast^5^	53.8 (256)	60.7 (91)	50.6 (165)*
School lunch^5^	54.0 (257)	60.7 (91)	50.9 (166)*
***Eating behaviors***			
Fruit and vegetables/day	3.5 ± 1.6	3.4 ± 1.7	3.5 ± 1.5
Sugar-sweetened beverages/day	1.7 ± 1.6	1.9 ± 2.0	1.6 ± 1.4**
Fast food meals/week	1.1 ± 1.1	1.4 ± 1.5	0.9 ± 0.9***^2^

^1 ^Comparisons were performed using *χ^2 ^*test (categorical variables) and *t*-test (continuous variables).

Statistically significant at level of: * *p *< 0.05 ** *p *< 0.01 *** *p *< 0.001

^2 ^Statistically significant after using Bonferroni correction for multiple comparison (Bonferroni-corrected *p *= 0.002).

^3 ^*n *= 585 due to missing data on self-reported height or weight.

^4 ^*n *= 117 who do not have a car available

^5 ^*n *= 476 households with ≥ 1 child

Adjusted linear regression estimates for the entire sample are shown in Table 2. Frequency of FFM and being a lone-parent household were independently associated with increased consumption of SSB; age and presence of at least one child were negatively associated with SSB. Being employed full-time and daily consumption of SSB were independently associated with increased frequency of FFM; age, receiving SNAP benefits, and being born in Mexico were associated with less frequent FFMs. In Table 3 nativity-stratified estimates for SSB indicate that increased frequency of FFM was independently associated with SSB among both U.S.- and Mexico-born women. The influence of age, presence of a child, or being a lone parent was significant among Mexico-born women only. The correlates of increased frequency of FFM are shown in Table 4. For U.S.-born women, SNAP participation was associated with lower FFM and SSB consumption with greater FFMs; and among Mexico-born women, full-time employment, being a lone parent, and SSB consumption were each independently associated with increased frequency of FFM.

**Table 2:** Association of Demographic Characteristics with Consumption of Sugar-Sweetened Beverages (SSB) and Frequency of Fast-Food Meals (FFM) among 599 Mexican-origin Women

Variable	SSB Consumption^1^ Coefficient (SE)	FFM Consumption^2^ Coefficient (SE)
Mexico-born	X	-0.43 (0.09)***
Age	-0.02 (0.01)***	-0.01 (0.01)*
≥ 1 Child in household	-0.45 (0.18)**	X
Lone parent household	0.33 (0.16)*	X
Fast food meals/week	0.36 (0.06)***	-
SSB consumption/daily	-	0.17 (0.03)***
SNAP	X	-0.27 (0.09)**
Employed full-time	X	0.27 (0.10)**

Intercept	2.44 (0.31)***	1.40 (0.18)***
Adjusted *R^2 ^*of model	0.122	0.154
Significance of *χ^2 ^*in model	< 0.0001	< 0.0001

^1 ^Dependent variable is consumption of regular soda or sugar-sweetened beverages (SSB) on an average day. All variables simultaneously entered; backward elimination of variables not statistically significant.

^2 ^Dependent variable is consumption of fast-food meals (FFM) during an average week. All variables simultaneously entered; backward elimination of variables not statistically significant.

X = dropped out of the model

Statistically significant at the level of: * *p *< 0.05 ** *p *< 0.01 *** *p *< 0.001

**Table 3:** Association of Demographic Characteristics and Frequency of Fast Food Meals (FFM) with Consumption of Sugar-Sweetened Beverages among 599 Mexican-origin Women, by Country of Birth

Variable	*U.S.-Born *(*n *= 194) Coefficient (SE)	*Mexico-born *(*n *= 405) Coefficient (SE)
Age	-0.02 (0.01)	-0.02 (0.01)***
≥ 1 Child in household	-0.01 (0.36)	-0.71 (0.20)***
Lone parent household	0.22 (0.31)	0.42 (0.19)*
FFM consumption/week	0.38 (0.09)***	0.32 (0.08)***

Intercept	2.04 (0.61)***	2.73 (0.35)***
Adjusted *R^2 ^*of model	0.098	0.116
Significance of *χ^2 ^*in model	< 0.0001	< 0.0001

Dependent variable is consumption of regular soda or sugar-sweetened beverages on an average day. All variables simultaneously entered.

Statistically significant at the level of: * *p *< 0.05 ** *p *< 0.01 *** *p *< 0.001

**Table 4:** Association of Demographic Characteristics with Weekly Consumption of Fast-Food Meals (FFM) among 599 Mexican-Origin Women, by Country of Birth

Variable	*U.S.-Born *(*n *= 194) Coefficient (SE)	*Mexico-born *(*n *= 405) Coefficient (SE)
Age	-0.01 (0.01)	-0.01 (0.01)
≥ 1 Child in household	0.12 (0.29)	-0.10 (0.13)
Lone parent household	0.09 (0.23)	0.25 (0.12)*
SNAP	-0.56 (0.23)**	-0.13 (0.09)
Employed full-time	0.27 (0.23)	0.26 (0.10)**
SSB consumption/day	0.22 (0.05)***	0.11 (0.03)***

Intercept	1.47 (0.48)**	1.01 (0.23)***
Adjusted *R^2 ^*of model	0.154	0.099
Significance of *χ^2 ^*in model	< 0.0001	< 0.0001

Dependent variable is weekly consumption of fast-food meals (FFM). All variables simultaneously entered.

Statistically significant at the level of: * *p *< 0.05 ** *p *< 0.01 *** *p *< 0.001

## Discussion

Although the incidence and prevalence of overweight and obesity have long reached critical levels and mirror increases in the consumption of SSB and FFM, especially among Hispanic adults and children, this is apparently the first study to examine the association of nativity with two less-healthy eating behaviors among Mexican women who reside in *colonias *along the Texas-Mexico border, where the population is primarily Mexican-origin and Spanish speaking individuals with similar spatial access to food stores and food service places [77]. Specifically, we examined the differences in SSB consumption and frequency of FFM between Mexican-origin women born in the United States and born in Mexico who were living in the same communities. This is also the first study that we are aware of that compared the influence of demographic characteristics on SSB and FFM consumption with Mexican-origin women born in the U.S. and in Mexico. There are three major findings related to nativity. First, U.S.-born women consumed more SSB and FFM than their Mexican-born counterparts. Second, in the combined sample and controlling for other population characteristics, being born in Mexico was independently associated with FFM (fewer FFM), but not with SSB. Third, in analyses stratified by nativity, FFM and SSB were associated with each other among both nativity groups. Possible reasons for observing an association between being born in Mexico and consuming fewer FFM include the higher proportion of Mexico-born with characteristics that represent lower socio-economic status, such as limited education, lower household income, employment status, and lack of transportation. The lack of association between nativity and SSB consumption may be explained by potentially distinct sources of SSB; U.S.-born may consume more SSB as purchased soft drinks while Mexico-born women may rely on *aguas frescas de frutas *(homemade fruit-flavored waters with added sugars).

Although similar proportions of U.S.- and Mexico-born Mexican women consumed at least one SSB each day or one FFM each week, U.S.-born women consumed a greater amount of SSB and greater number of FFM than their Mexico-born counterparts. Food and food habits have been linked to various measures of acculturation - single-item measures (e.g., country of birth or language), acculturation scores, and food-based assessments [86]. Although other studies have found years in the United States and language spoken to be associated with consumption of sugar beverages, this is apparently the first study to introduce the dimension of country of birth as an influence on SSB consumption [33], especially in the context of the predominantly Mexican-origin, Spanish language communities along the Texas-Mexico border. As previously mentioned, one study of San Francisco Bay Area Hispanic women documented a relationship between country of birth and fast-food consumption [40]. Notable demographic differences observed between Mexican women born in Mexico and those born in the U.S. suggests that country of birth may serve as a proxy measure for limited education, very low household income, being married, larger household composition, not having a car available during the day, and employment status outside the home. The results confirm prior reviews and research articles suggesting that less acculturation (e.g., country of birth) is associated with healthier diets [33,34,87]. This study is unique in that Hispanic subgroups (U.S.-born and Mexico-born) experience similar contextual exposures, such as density of Mexican population, *colonia *population characteristics, and locational access to food sources, which are considered a major influence on the acculturation process [33]. Stratified multiple variable regression analyses confirmed nativity differences and similarities in the influence of sample characteristics and eating behaviors on SSB and FFM consumption.

The observation of co-occurring unhealthy eating and drinking behaviors such as higher consumption of SSB and FFM has been reported previously [24,75]. Several studies document the association between both unhealthy dietary behaviors and adverse health incomes, including weight gain and obesity risk [17,23], cardiometabolic risk [22,88,89], and type 2 diabetes [17]. This study adds to the paucity of research documenting SSB and FFM consumption among Mexican-origin women [16,27,39], a population identified as at-risk for developing diet-related chronic-diseases [16,27]. At present, Mexican-origin women have the highest rates of obesity in the U.S. and in Mexico [1,2,26].

There are a number of strengths to this study. First, this is a large study of hard-to-reach Mexican women in border *colonias*. This population is of increasing national importance because such *colonias *can be considered an archetype for the new destination Mexican immigrant communities that are now found in great numbers throughout the continental U.S. Second, there are few studies describing dietary factors associated with obesity, including consumption of SSB and FFM among a sample of Mexican-origin women [16,27,39]. Previous studies with men and women of Mexican-origin have included at least one of these unhealthy dietary behaviors, but only in the context of general dietary patterns [13-15]. Understanding risk markers for obesity in Mexican-origin women is a major public health focus on both sides of the border and a bi-national priority [5,7,19], which bolsters the importance of this study. A third strength is the identification of country of birth as a single-item measure of acculturation in a Mexican population, where Spanish is the predominant language.

There are several limitations to this study that warrant mention. First, the self-reported measures of SSB and FFM consumption may underreport actual frequency and amount consumed each day, which limits our ability to determine caloric intake from SSB or FFM. These measures of SSB and FFM, which did not identify a specific time frame, provided incomplete descriptions and did not specify culturally-appropriate SSB such as *aguas frescas de frutas*. As others have suggested, there is a need for specific prompts to better capture intakes of SSB and FFM [25,69,90]. Second, data were not available to describe seasonal variation in consumption of SSB and FFM. Third, data were not available to document type and amount of FFM consumed. Fourth, there were no additional measures of acculturation such as duration of residence in the U.S. Finally, the cross-sectional nature of this study prevents a temporal determination of predictors of consumption of SSB and FFM.

Despite these limitations, this research is highly relevant and timely given that the largest growing segment of the U.S. population is of Mexican origin and the concomitant increase in the number of new destination immigrant communities in interior and rural destinations including Iowa, North Carolina, and Tennessee [6,7,9,91,92]. *Colonias *are considered the archetype for new destination communities of Mexican-origin immigrants. Similar to new destination immigrant communities, *colonias *are smaller, more dispersed communities comprised of a disproportionately poor population of immigrants and their families with limited access to the kinds of resources necessary for facilitating economic and social mobility in a region characterized by adverse social conditions [9,93]. A distinguishing characteristic of *colonias *is that *colonias *are not "new" and now include both native and immigrant residents of Mexican-origin. Given consideration of the prototypical character of *colonias*, these findings have implications for dietitians developing interventions focused on the reduction of FF and SSB meals among Mexican-origin residents in rapidly growing new destination communities.

## Conclusions

This study provides key findings on the importance of considering nativity in consumption of SSB and FFM. U.S.-born women consumed more SSB and FFM than Mexican-born counterparts in the same areas of *colonias*. Further, being born in Mexico was independently associated with consuming fewer FFM, but not associated with SSB. Finally, FFM and SSB were associated with each other among both nativity groups. Future research should focus on expanding our understanding of the meaning of nativity among individuals who share common contextual factors, but likely differ in life-course experiences and the resources needed to transition into a new place. Nativity may function as a proxy for acculturation and indicate the extent that immigrants adopt behaviors from their new environment. However, nativity could also indicate limited accessibility to resources such as food/nutrition assistance programs, transportation, and proper documentation. Additional measures should be considered such as educational and occupational background, resilience and access to a range of resources, migration history, documentation status, dietary acculturation, and life-course experiences, which may better explain heterogeneity in health outcomes among Hispanic subgroups [92,93].

## Competing interests

The authors declare that they have no competing interests.

## Authors' contributions

JRS developed the original idea for assessing sugar beverages and fast food. JRS worked on the development of the instrument and the protocol for collection of data. JRS, CMJ, and WRD wrote the first draft of the paper. JRS, CMJ, and WRD read and approved the final manuscript.
